# Healthy sleep practices for shift workers: consensus sleep hygiene guidelines using a Delphi methodology

**DOI:** 10.1093/sleep/zsad182

**Published:** 2023-07-10

**Authors:** Alexandra E Shriane, Gabrielle Rigney, Sally A Ferguson, Yu Sun Bin, Grace E Vincent

**Affiliations:** Appleton Institute, School of Health, Medical and Applied Sciences, Central Queensland University, Adelaide, SA, Australia; Appleton Institute, School of Health, Medical and Applied Sciences, Central Queensland University, Adelaide, SA, Australia; Appleton Institute, School of Health, Medical and Applied Sciences, Central Queensland University, Adelaide, SA, Australia; Charles Perkins Centre, University of Sydney, Sydney, NSW, Australia; Northern Clinical School, Sydney Medical School, Faculty of Medicine and Health, University of Sydney, Sydney, NSW, Australia; Appleton Institute, School of Health, Medical and Applied Sciences, Central Queensland University, Adelaide, SA, Australia

**Keywords:** shift work, fatigue management, Delphi, recommendations, occupational health

## Abstract

**Study Objectives:**

The unique requirements of shift work, such as sleeping and working at variable times, mean that current sleep hygiene guidelines may be inappropriate for shift workers. Current guidelines may also contradict fatigue management advice (e.g. advising against daytime napping). The present study utilized a Delphi methodology to determine expert opinion regarding the applicability of current guidelines for shift workers, the appropriateness of the term “sleep hygiene,” and develop tailored guidelines for shift workers.

**Methods:**

The research team reviewed current guidelines and existing evidence to draft tailored guidelines. Seventeen individual guidelines, covering sleep scheduling, napping, sleep environment, bedtime routine, substances, light exposure, diet, and exercise were drafted. Experts from sleep, shift work, and occupational health fields (*n* = 155) were invited to review the draft guidelines using a Delphi methodology. In each round, experts voted on individual guidelines, with 70% agreement considered consensus. Where consensus was not reached, written feedback from experts was discussed and incorporated into subsequent iterations.

**Results:**

Of the experts invited, 68 (44%) agreed to participate, with 55 (35%) completing the third (final) round. Most experts (84%) agreed that tailored guidelines were required for shift workers. Consensus was reached on all guidelines after three rounds. One additional guideline (sleep inertia) and an introductory statement were developed, resulting in a final set of 18 individual guidelines, termed “healthy sleep practices for shift workers.”

**Conclusions:**

This is the first study to develop tailored sleep hygiene guidelines for shift workers. Future research should investigate the acceptability and effectiveness of these guidelines amongst shift workers.

## Graphical Abstract

**Graphical Abstract:**
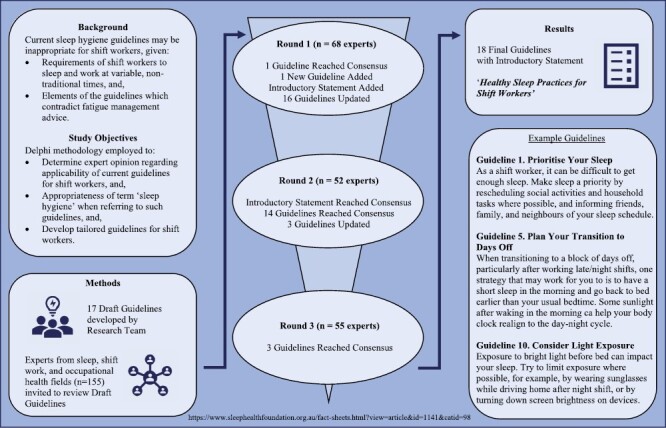

**Statement of Significance**

Sleep hygiene guidelines, as currently written, may be impractical for shift workers, given the irregularity of their sleep/wake schedules. In addition, current sleep hygiene guidelines may contradict fatigue management advice (i.e. by advising against caffeine consumption and daytime napping). Furthermore, outcomes from previous works suggest that 85% of shift workers have limited, if any, understanding of “sleep hygiene” as a concept. Given the significantly higher rates of inadequate sleep amongst shift workers, and the wide range of associated adverse health and well-being effects, appropriate sleep hygiene guidelines for shift workers are critical. This study is the first to present expert-developed, consensus-based sleep hygiene guidelines for use by shift workers.

## Introduction

Sleep hygiene describes a range of lifestyle and environmental factors that can act to optimize sleep. Sleep hygiene guidelines were originally developed by Dr Peter Hauri as a management tool for adult insomnia [1] based on the assumption that the components included within the guidelines impacted sleep, and that improving these components would improve sleep outcomes [2]. Since the introduction of sleep hygiene guidelines in the late 1970’s [1] (see Table 1 for original guidelines), numerous sets of sleep hygiene guidelines have built on the original, often containing slight variations in language or advice, representing the ever-expanding evidence base [3,4]. However, the overarching components of sleep hygiene guidelines have remained relatively unchanged, and usually address sleep schedule, naps, substances (caffeine, nicotine, and alcohol), bedtime activities, bedroom environment, diet, and exercise [5]. While sleep hygiene guidelines have since been shown to offer minimal improvement for adult insomnia as a standalone intervention [6, 7], the use of sleep hygiene guidelines has been demonstrated to improve a range of sleep outcomes across some chronic conditions which negatively impact sleep, such as psychological disorders [8] and genetic conditions [9], and those requiring acute, temporary intervention (e.g. chemotherapy) [10]. Furthermore, there is evidence that the use of sleep hygiene guidelines can improve sleep outcomes across a range of populations, including children and adolescents [11, 12], adults [13, 14], older adults [15], and athletes [16, 17]. Despite being originally intended for use in clinical populations, sleep hygiene guidelines are now also utilized as a tool for improving the general sleep health of non-clinical groups [18]. However, certain populations experience unique challenges that are not accounted for by current sleep hygiene guidelines (e.g. not being able to go to bed and wake at the same time each day). Individuals who engage in shift work are one such population.

**Table 1.** Sleep Hygiene Guidelines as Originally Presented by Dr Peter Hauri [1]

Sleep as much as needed to feel refreshed and healthy during the following day, but not more. Curtailing time in bed a bit seems to solidify sleep; excessively long times in bed seem related to fragmented and shallow sleep.
A regular arousal time in the morning seems to strengthen circadian cycling and to finally lead to regular times of sleep onset.
A steady daily amount of exercise probably deepens sleep over the long run, but occasional one-shot exercise does not directly influence sleep during the following night.
Occasional loud noises (e.g. aircraft flyovers) disturb sleep even in people who do not awaken because of the noises and cannot remember them in the morning. Sound attenuating the bedroom might be advisable for people who have to sleep close to excessive noise.
Although an excessively warm room disturbs sleep, there is no evidence that an excessively cold room solidifies sleep, as has been claimed.
Hunger may disturb sleep. A light bedtime snack (especially warm milk or similar drink) seems to help many individuals.
An occasional sleeping pill may be of some benefit, but the chronic use of hypnotics is ineffective at most and detrimental in some insomniacs.
Caffeine in the evening disturbs sleep, even in persons who do not feel it does.
Alcohol helps tense people to fall asleep fast, but the ensuing sleep is then fragmented.
Rather than trying harder and harder to fall asleep during a poor night, switching on the light and doing something else may help the individual who feels angry, frustrated, or tense about being unable to sleep.

Shift work requires individuals to engage in work outside of traditional working and waking hours (i.e. outside the “normal” Monday–Friday, 09:00 am–05:00 pm routine), and incorporates variation in work periods (extended, condensed, and split), and work schedules (rotating, fixed) [19]. Engagement in shift work has steadily increased in developed countries since the Industrial Revolution [20], with shift workers now estimated to comprise up to one-quarter of the workforce in developed countries [21]. Despite contributing positively to productivity and economic growth, shift work can take a significant toll on the health and well-being of the individual worker [22]. A multitude of complex mechanisms are responsible for the negative health and well-being impacts of shift work, including light exposure at inappropriate times, shortened sleep periods, and nontraditional wake and rest times, with these factors all impacting one common element: sleep [19].

Shift work requires individuals to be awake and active at nontraditional times, on a repeated and ongoing basis. This pattern of behavior leads to disruptions in circadian rhythms, the 24-hour rhythms that regulate a range of bodily functions (e.g. hormone secretion, appetite, temperature control, etc.) [23]. Persistent disruptions in these rhythms can result in circadian misalignment, whereby circadian rhythms are no longer aligned with the diurnal (day/night) cycle [24]. Circadian misalignment results in difficulty initiating and maintaining sleep, with shift workers more likely to obtain insufficient sleep (i.e. less than 7 hours of sleep per 24 hours) and experience insomnia symptoms or excessive sleepiness associated with their work schedules (i.e. shift work disorder) than day workers [25, 26]. Beyond sleep outcomes, circadian misalignment increases shift workers’ risks of developing diabetes [27], coronary artery disease [28], obesity [29], hypertension [30], and a range of cancers [19]. As a result of their working arrangements, shift workers are also more likely to experience psychological distress [31], decreased job satisfaction [32], and workplace errors and accidents [33]. Considering the significant health and well-being impacts of shift work, it is not uncommon for shift workers to seek information or medical advice on how to manage the consequences of shift work, and when they do, sleep hygiene guidelines may be provided [34, 35].

As a result of the unique conditions of shift work, some elements of current sleep hygiene guidelines may be ineffective or impractical for shift workers. For example, current guidelines usually recommend limiting napping during daytime hours, and avoiding caffeine in the 6–8 hours before bed [18, 36], despite both practices being considered safe and effective fatigue management strategies for shift workers [37–39]. Furthermore, current sleep hygiene guidelines recommend maintaining regular sleep and wake times [18, 40], which is impractical for most shift workers, particularly those who work variable or rotating rosters. More generally, current sleep hygiene guidelines are constructed with language that describes diurnal sleep–wake patterns, and fail to address the unique challenges faced by shift workers, such as incorporating health behaviors (e.g. exercise, pre-sleep wind-down) around shift work commitments, or sleeping during the day with increased noise and light.

The advice included within current sleep hygiene guidelines is one aspect that requires review for use amongst shift workers, but the terminology used in referring to such advice also needs consideration. Peter Hauri himself, who is credited with first codifying sleep hygiene concepts into a set of guidelines, admits never having liked the term “sleep hygiene” [41]. Recent evidence suggests that the term is not well understood by shift workers—approximately half of shift workers are familiar with the term “sleep hygiene,” and only 11%–15% of shift workers reported understanding the term “extremely well” [42, 43]. As such, the content of current sleep hygiene guidelines, as well as the terminology used in describing them, needs to be reevaluated for use amongst shift workers. Therefore, the aims of this study are to:

1. Determine the opinion of experts on the applicability of current sleep hygiene guidelines for shift workers, and,
2. Determine the appropriateness of “sleep hygiene” as a term to describe a range of lifestyle and environmental factors that can act to optimize sleep, and,
3. Utilizing a Delphi methodology, develop a set of tailored sleep hygiene guidelines for shift workers.

## Methods

### Delphi methodology

A Delphi methodology was employed to develop tailored sleep hygiene guidelines for shift workers. A Delphi methodology is used to derive consensus on topics where consensus does not currently exist, and/or make a decision based on the opinion of a panel of experts [44]. This method invites participants with appropriate expertise in relevant fields to participate in iterative rounds of questionnaires, with their responses and feedback informing subsequent rounds [44]. There is no prescriptive number of expert participants required [45], however, a range of recent sleep-related Delphi studies utilized between 15 and 75 expert participants [46–51]. Furthermore, there is no definitive number of rounds, instead, rounds usually continue until consensus on the relevant outcome/s has been reached [50], with 2–4 rounds being most common [52]. Finally, there is no defined point at which consensus is reached in a round; however, 70% agreement on items is widely accepted as having reached consensus [51].

### Procedure and measures

Ethics approval for this study was obtained from the Human Research Ethics Committee, Central Queensland University, Australia (reference number 0000022810). Prior to commencing the study, a draft set of sleep hygiene guidelines for shift workers was developed by the research team (AES, GR, SAF, YSB, and GEV). The draft sleep hygiene guidelines for shift workers were constructed based on current guidelines, fatigue management strategies for shift workers, and research investigating shift worker understanding of “sleep hygiene” as a concept and their engagement with current guidelines (e.g. consumption of caffeine, maintenance of a regular sleep schedule) [42, 43, 53]. The draft sleep hygiene guidelines for shift workers are presented in Supplementary Table 1, and encompass the following themes, with examples of supporting evidence for such themes: ensuring sufficient sleep duration [54–59], attempting to sleep and wake at the same time/s [60–62], engaging in a relaxing bedtime routine [63–66], transitioning to days off work [67–72], napping for fatigue management [73–80], maintaining a comfortable sleep environment [81–86], managing exposure to light [87–92], managing caffeine consumption [18, 93, 94], limiting alcohol and nicotine [95–98], considering medication use [99–103], considering food and fluid intake [104–108], engaging in regular exercise [109–111], and managing sleep problems [112–114]. No individuals outside of the research team had access to, or information regarding, the draft guidelines prior to the study commencing, to avoid any inadvertent bias for those who may become participants.

The research team compiled a list of experts (*n* = 155), who were identified based on their knowledge of, and contribution to, the relevant fields (sleep, shift work, and occupational health). Experts were invited if they (1) were either author or coauthor of peer-reviewed publications in Q1 and Q2 journals (based on Web of Science journal impact factor ranking) on relevant topics (e.g. health impacts of shift work, behavioral interventions to improve sleep outcomes etc.), or (2) had experience working in a relevant industry setting (e.g. shift worker health and safety, sleep clinician).

Experts were contacted by email and invited to participate in a series of brief, iterative online questionnaires, expected to take 5–15 minutes each to complete. This initial email advised experts that their participation would be blinded to other experts, and that their responses and feedback would be incorporated into subsequent questionnaire rounds, until consensus was reached on all individual guidelines. To facilitate recruitment, experts were encouraged to circulate the invitation amongst appropriately qualified colleagues, which resulted in two additional expert participants.

The round 1 questionnaire first collected demographic information about the experts, including gender, geographical location, field of expertise, current role, and years of experience. Experts were then asked their opinion on the terminology (i.e. the appropriateness of the term “sleep hygiene” in describing a range of lifestyle and environmental factors that can optimize sleep), and content (i.e. the appropriateness of current guidelines for shift workers) of sleep hygiene guidelines as presently written. Both questions were answered on a 5-point Likert scale (from “strongly disagree*”* to “strongly agree*”*), while in relation to terminology, experts were also provided with an opportunity to suggest alternate terminology, with these terms then presented to experts in subsequent rounds for voting on preference. Following these contextual questions, experts were asked to review the draft sleep hygiene guidelines for shift workers as developed by the research team, and determine to what extent they agreed with each individual guideline being included as written. Experts again responded on a 5-point Likert scale, which included rankings from “strongly disagree” to “strongly agree.” If an expert responded with “strongly disagree,” “disagree,” or “neither agree nor disagree,” they were prompted to provide free-text feedback, describing how the individual guideline should be modified. A response of “agree” or “strongly agree” was considered support for the individual guideline being included as written. A consensus cutoff point was set at 70% for each individual guideline (i.e. 70% of experts selected “agree” or “strongly agree”). Once consensus was reached on all individual guidelines, experts were offered the opportunity to place them in the most appropriate order to establish a final set of guidelines.

**Figure 1.**
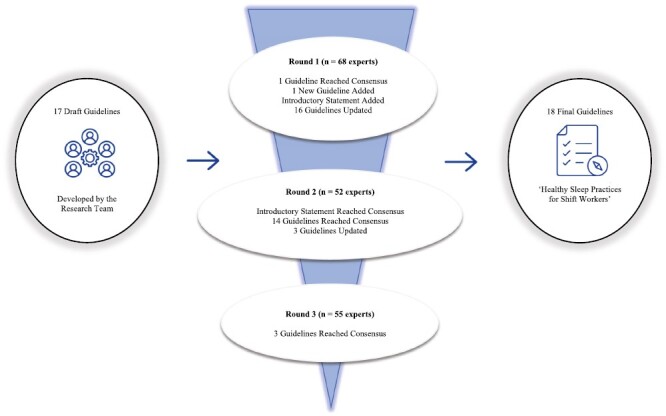
Development of Healthy Sleep Practices for Shift Workers using a Delphi Methodology.

The round 1 questionnaire was open for four weeks. Following this, the research team convened to review the outcomes and discuss the free-text responses. These roundtable discussions involved all members of the research team reviewing all free-text feedback provided by experts when they had selected Likert scale rating responses of “strongly disagree,” “disagree,” or “neither agree nor disagree” in response to a guideline being included as written. In reviewing the free-text feedback, those responses which articulated themes being echoed by multiple experts were considered to mandate action by the research team, either in the form of guideline modification, guideline removal, or new guideline development. The same roundtable process was then repeated following questionnaire rounds 2 and 3, after which time consensus had been reached on all individual guidelines. Table 2 outlines the point at which consensus was reached for each individual guideline, while Figure 1 demonstrates the Delphi methodology as used in the study.

**Table 2.** Consensus Outcomes for Healthy Sleep Practices for Shift Workers

Guideline	Round consensus reached	Disagree with inclusion as written after round 3 (%)		
	(Round & %)	*Neutral*	*Disagree*	*Strongly disagree*
Introductory statement^*^	Round 2(92.1)	5.9	2.0	0
Sleep prioritization	Round 2(92.1)	5.9	2.0	0
Sleep duration	Round 2(78.4)	9.8	11.8	0
Sleep schedule	Round 3(72.7)	16.4	10.9	0
Bedtime routine	Round 2(82.3)	11.8	5.9	0
Transition to days off	Round 3(81.8)	16.4	1.8	0
Napping	Round 3(83.6)	12.7	3.7	0
Sleep inertia^*^	Round 2(84.3)	7.8	5.9	2.0
Sleep environment	Round 2(90.2)	7.8	2.0	0
Bed use	Round 2(80.4)	15.7	3.9	0
Light	Round 2(70.6)	13.7	15.7	0
Caffeine	Round 2(76.5)	15.7	7.8	0
Nicotine	Round 1(73.5)	22.1	4.4	0
Alcohol	Round 2(92.2)	3.9	3.9	0
Medication use	Round 2(72.5)	13.7	11.8	2.0
Food intake	Round 2(80.4)	13.7	5.9	0
Fluid intake	Round 2(84.3)	9.8	5.9	0
Exercise	Round 2(78.4)	13.7	7.9	0
Sleep problems	Round 2(90.2)	5.9	3.9	0

^*^Denotes items that were developed during Delphi rounds as a result of expert feedback.

Throughout, the research team was in agreement about the decisions made during the roundtable discussions (e.g. the creation of a new guideline, the modification to wording of an existing guideline etc.); however, had disagreements arisen, the research team was comprised of an odd number of members to facilitate a “majority rules” process to achieve resolution.

## Results

### Expert panel demographics

Of the 155 experts invited to participate in the study, 68 (44%) consented and completed round 1, with 52 of those (34%) completing round 2, and 55 (35%) completing round 3. All experts who had consented to participate were invited to complete each round, even if they had missed the deadline for completion of the previous round, resulting in three additional participants in round 3 compared to round 2.

Demographic data are presented below in Table 3. Almost two-thirds (65%) of experts identified as female, with most residing in Australia (47%) or the United States (22%). Most experts (65%) described their job title as Professor, Associate/Assistant Professor, or Postdoctoral Researcher, with over half (55%) indicating that their expertise was in sleep research, shift work, chronobiology, and sleep disorders, with 17.7 mean years (±9.4 SD) of expertise. Given the relatively small field from which participants were recruited, and the nature of work conducted at the authors’ institution, the research team was cognizant of avoiding potential overrepresentation of individuals from their own institution in the participant pool. Ten (6.5%) of the one hundred and fifty-five experts invited to participate were from the same institution as the authors, of which six completed round 1 (8.8% of participants in this round), four completed round 2 (7.7%), and four completed round 3 (7.3%).

**Table 3.** Demographics of Invitees and Expert Participants

	Invitees^*^(%)*N* = 155	Round 1 experts (%)*N* = 68	Round 2 experts (%)*N* = 52	Round 3 experts (%)*N* = 55
Gender				
Female	82 (52.9%)	44 (64.7%)	35 (67.3%)	38 (69.1%)
Male	73 (47.1%)	24 (35.3%)	17 (32.7%)	17 (30.9%)
Country of residence				
Australia	71 (45.8%)	33 (47.1%)	27 (51.9%)	27 (49.3%)
United States of America	43 (27.7%)	15 (22.0%)	11 (21.1%)	12 (21.9%)
Canada	7 (4.5%)	3 (4.4%)	1 (1.9%)	2 (3.6%)
Netherlands	7 (4.5%)	2 (2.9%)	2 (3.9%)	2 (3.6%)
England	5 (3.2%)	1 (1.5%)	1 (1.9%)	1 (1.8%)
Sweden	5 (3.2%)	2 (2.9%)	2 (3.9%)	2 (3.6%)
Finland	4 (2.6%)	2 (2.9%)	2 (3.9%)	2 (3.6%)
Germany	3 (1.9%)	3 (4.4%)	1 (1.9%)	1 (1.8%)
Norway	3 (1.9%)	1 (1.5%)	0 (0%)	0 (0%)
South Africa	2 (1.3%)	2 (2.9%)	2 (3.9%)	2 (3.6%)
Thailand	1 (0.7%)	1 (1.5%)	1 (1.9%)	1 (1.8%)
France	1 (0.7%)	1 (1.5%)	0 (0%)	1 (1.8%)
Italy	1 (0.7%)	1 (1.5%)	1 (1.9%)	1 (1.8%)
New Zealand	1 (0.7%)	1 (1.5%)	1 (1.9%)	1 (1.8%)
Singapore	1 (0.6%)	0 (0%)	0 (0%)	0 (0%)
Primary job role ^#^				
Researcher	104 (67.1%)	51 (75.0%)	40 (76.9%)	42 (76.3%)
Health professional	33 (21.3%)	12 (17.6%)	9 (17.3%)	9 (16.4%)
Industry expert	18 (11.6%)	5 (7.4%)	3 (5.8%)	4 (7.3%)
Job title (self-described)				
Professor or A/professor		25 (36.8%)	18 (34.7%)	20 (36.4%)
Postdoctoral researcher		19 (28.0%)	14 (27.0%)	14 (25.5%)
PhD candidate		4 (5.9%)	4 (7.7%)	4 (7.3%)
Lecturer		3 (4.4%)	3 (5.8%)	3 (5.5%)
Specialist		3 (4.4%)	3 (5.8%)	3 (5.5%)
Consultant		2 (2.9%)	0 (0%)	1 (1.8%)
Director		2 (2.9%)	2 (3.8%)	2 (3.6%)
Physician		2 (2.9%)	1 (1.9%)	1 (1.8%)
Psychologist		2 (2.9%)	2 (3.8%)	2 (3.6%)
Scientist		2 (2.9%)	2 (3.8%)	2 (3.6%)
Epidemiologist		1 (1.5%)	0 (0%)	0 (0%)
Dean		1 (1.5%)	1 (1.9%)	1 (1.8%)
Division Head		1 (1.5%)	1 (1.9%)	1 (1.8%)
Trainer		1 (1.5%)	1 (1.9%)	1 (1.8%)
Years worked in area of expertise				
0–4		2 (2.9%)	1 (1.9%)	1 (1.8%)
5–9		9 (13.2%)	8 (15.4%)	8 (14.6%)
10–14		13 (19.1%)	9 (17.3%)	10 (18.2%)
15–19		19 (28.0%)	13 (25.0%)	13 (23.6%)
20–24		11 (16.2%)	10 (19.2%)	11 (20.0%)
25–29		4 (5.9%)	3 (5.8%)	3 (5.5%)
30–34		7 (10.3%)	5 (9.6%)	6 (10.9%)
35–39		0 (0%)	0 (0%)	0 (0%)
40–44		1 (1.5%)	1 (1.9%)	1 (1.8%)
45–50		2 (2.9%)	2 (3.9%)	2 (3.6%)
Mean (years)		17.7	18.8	18.9

^*^Invitee demographics are based on publicly available information accessed by the authors when identifying experts for participation, and therefore lack specific information on job title and years of expertise.

^#^Participants were required to select one primary job role, with this result therefore not indicative of individuals who are employed in multiple contexts (i.e. a clinician-researcher may be represented as either a clinician or researcher, dependent on the primary job role they identified). Primary job role for invitees is based on the author's deductions from publicly available information.

### Round 1 results: acceptability of current guidelines and terminology

In response to questions regarding the applicability of the current sleep hygiene guidelines to shift workers, most experts (71%) indicated that they “disagreed” or “strongly disagreed” that the current guidelines were appropriate for use amongst shift workers. Furthermore, most experts (84%) responded that they “agreed” or “strongly agreed” that tailored sleep hygiene guidelines were required for this group. Regarding the appropriateness of the term “sleep hygiene,” approximately half of experts (57%) “agreed” or “strongly agreed” that it was appropriate in referring to lifestyle and environmental factors that can act to optimize sleep, while over one-third (39%) “disagreed” or “strongly disagreed,” and few experts (4%) “neither agreed nor disagreed.” In follow-up, experts were asked to suggest alternate terminology that could replace “sleep hygiene,” with the most common responses, “healthy sleep practices” (15%), “sleep-promoting behaviors” (12%), “healthy sleep behaviors” (12%), “sleep habits” (9%), and “sleep behaviors” (9%), fed-back to experts in round 2.

### Round 1 results: review of draft sleep hygiene guidelines for shift workers

Upon closure of the round 1 questionnaire, one guideline had reached consensus (on nicotine), with all other guidelines progressing to round 2. As a result of the free-text responses from experts when providing feedback on the draft guidelines, an introductory statement and one additional guideline (on sleep inertia) were developed and added to the questionnaire for expert review in round 2.

### Round 2 results: preferred terminology and outstanding sleep hygiene guidelines

In round 2, one follow-up question was asked regarding expert preference on alternate terminology for “sleep hygiene,” based on the five most common terms suggested in round 1, with “healthy sleep practices” being the most preferred (Table 4). Sixteen of the draft guidelines, an introductory statement, and one additional guideline were included for review in round 2. Fourteen individual guidelines and the introductory statement reached consensus in round 2, with three individual guidelines (on sleep scheduling, transitioning to days off, and napping) reviewed and updated for inclusion in round 3.

**Table 4.** Expert Feedback on Alternate Terminology to describe “Sleep Hygiene”

	No. of participants (%)*N* = 52
Healthy sleep practices	28 (53.8%)
Healthy sleep behaviors	16 (30.8%)
Sleep promoting behaviors	8 (15.4%)
Sleep behaviors	0 (0%)
Sleep habits	0 (0%)

### Round 3 results: outstanding sleep hygiene guidelines

In round 3, the three remaining individual guidelines were included, and subsequently, reached consensus. When presented with the opportunity to rearrange the order in which the guidelines were presented, half of the experts (51%) elected to maintain the order in which the research team had placed them. Those experts that did choose to reorder the guidelines were able to position each individual guideline in their preferred place (i.e. first, second, third etc.), with the majority of experts placing each individual guideline into the same place that the research team had presented them. Further detail on expert-preferred guideline placement is described in Table 5.

**Table 5.** Expert Preference on Order of Healthy Sleep Practices for Shift Workers

	Preferred Placement (%)*N* = 27^*^
Prioritize your sleep	1st (96.3%)
Aim for 7–9 hours sleep per 24 hours	2nd (70.4%)
Develop a sleep schedule	3rd (88.9%)
Develop a bedtime routine	4th (81.5%)
Plan your transition to days off	5th (55.6%)
Use napping as a helpful tool	6th (74.1%)
Consider sleep inertia	7th (70.4%)
Create a comfortable sleep environment	8th (48.2%)
Use your bed for sleep and intimacy	9th (59.3%)
Consider light exposure	10th (66.7%)
Consider caffeine intake	11th (63.0%)
Consider nicotine consumption	12th (55.6%)
Consider alcohol intake	13th (63.0%)
Be mindful of medication	14th (55.6%)
Consider food intake	15th (63.0%)
Consider fluid intake	16th (74.1%)
Engage in regular exercise	17th (74.1%)
Develop strategies for sleep problems	18th (51.9%)

^*^In round 3 questionnaire, *n* = 28 experts elected to not rearrange the order of healthy sleep practices for shift workers, therefore, these results represent the preferences of those experts who did opt to rearrange them.

Review of free-text responses determined no additional guidelines or statements required development, and as such, no further rounds were necessary, and the Delphi methodology was deemed complete. Table 6 presents the final version of the guidelines.

**Table 6.** Healthy Sleep Practices for Shift Workers

**Introductory Statement** As a shift worker, it can be difficult to get enough good quality sleep, which can impact health, well-being, job performance, and safety. The following guidelines are designed to provide shift workers with advice on healthy sleep practices, which can improve sleep during rostered periods of work.These guidelines are based on scientific evidence and offer strategies that will work for most people. However, it is important to remember that everyone is different. Please use them as a guide, and incorporate them based on your shift schedule, lifestyle, commitments, etc.If you have any concerns or queries about your sleep or managing the effects of shift work, it’s important to seek advice from a health professional. Your general practitioner/primary care provider is a good place to start, while sleep physicians and sleep psychologists can help with tailored treatments.
**Guideline 1. Prioritize your sleep** As a shift worker, it can be difficult to get enough sleep. Make sleep a priority by rescheduling social activities and household tasks where possible, and informing friends, family, and neighbors of your sleep schedule.
**Guideline 2. Aim for 7–9 hours of sleep per 24 hours** Your individual sleep needs may differ, but research shows that 7–9 hours is the amount of sleep most healthy adults require. This may be achieved as one single sleep period, or as a main sleep supplemented by a shorter sleep(s). Keep in mind that this is total time spent asleep, not just time in bed.
**Guideline 3. Develop a sleep schedule** Your sleep schedule should be based on your roster and lifestyle. Try to maintain a similar sleep schedule for each shift type (e.g. Bedtime A for day shifts, Bedtime B for afternoon shifts, etc.), remembering to allow a sufficient opportunity for sleep (i.e. 7–9 hours total over 24 hours).
**Guideline 4. Develop a bedtime routine** Find activities that help you wind-down and feel relaxed, and consistently engage in these activities before bed, ideally in a dimly lit and quiet environment. This is particularly beneficial if you have trouble falling asleep.
**Guideline 5. Plan your transition to days off** ^*^ When transitioning to a block of days off, particularly after working late/night shifts, one strategy that may work for you is to have a short sleep in the morning and go back to bed earlier than your usual bedtime. Some sunlight after waking in the morning can help your body clock realign to the day-night cycle.
**Guideline 6. Use napping as a helpful tool** Short naps (15–20 minutes) can boost alertness and performance, while longer naps (90 minutes) can reduce sleep debt. Naps less than 15 minutes may be too short to be beneficial, while naps longer than 20 minutes may make you more likely to experience sleep inertia (*see guideline 7*). Keep in mind that longer naps should be avoided in the 4–6 hours before your main sleep as they may make it more difficult to fall asleep. Ideally, nap in a quiet, dark, and cool environment for best sleep quality.
**Guideline 7. Consider sleep inertia** ^*^ After waking, shift workers may experience sleep inertia - a period of grogginess, where alertness and performance are impaired. This feeling typically lasts 15–30 minutes after waking but can last up to 2 hours. It is important to avoid high-risk tasks (e.g. driving, operating machinery) during this time.
**Guideline 8. Create a comfortable sleep environment** Aim to sleep somewhere that is:Comfortably cool: 16–20ºC/ 60–68ºF with adequate ventilation.Dark: block out as much light as possible (e.g. use appropriate window furnishings, wear an eye mask).Quiet: block out as much noise as possible (e.g. close doors and windows, use ear plugs, switch off devices). Some people find white noise helpful.
**Guideline 9. Use your bed for sleep and intimacy** Use your bed for sleeping and intimacy only, if possible. Avoid mentally stimulating activities in bed (e.g. playing video games, working on a laptop), and be mindful of sharing your bed with others (e.g. pets) who may disturb your sleep.
**Guideline 10. Consider light exposure** Exposure to bright light before bed can impact your sleep. Try to limit exposure where possible, for example, by wearing sunglasses while driving home after night shift, or by turning down screen brightness on devices.
**Guideline 11. Consider caffeine intake** Caffeine can help to improve alertness and performance before and during your shift. However, the effects of caffeine can last for several hours, often longer than you think, and vary greatly between people. Keep in mind that caffeine too close to your bedtime may impact your sleep.
**Guideline 12. Consider nicotine consumption** Avoid nicotine entirely, or limit nicotine intake in the 6 hours before bed.
**Guideline 13. Consider alcohol intake** Avoid alcohol as part of your bedtime routine. Some people feel that alcohol helps them fall asleep. However, drinking alcohol close to bedtime, even in small amounts, negatively impacts your sleep quality.
**Guideline 14. Be mindful of medication** Medications can impact sleep. Some medications have stimulant effects, and ideally, shouldn’t be taken near bedtime. Some natural substances, like melatonin, can be helpful for shift workers experiencing sleep problems. Sleep-inducing medications (i.e. sleeping tablets) should usually only be used for short-term or intermittent relief of sleep problems. Always consult a healthcare professional regarding medication use and its impact on your sleep.
**Guideline 15. Consider food intake** Where possible, limit food intake during night shifts, and if you do eat, opt for smaller, lighter meals. Don’t go to bed hungry, as this may negatively impact sleep, but choose a lighter meal before bed that won’t cause indigestion or discomfort.
**Guideline 16. Consider fluid intake** It’s important to maintain hydration by drinking plenty of water; however, avoid too much fluid before bed, as this may lead to sleep disturbances to use the toilet.
**Guideline 17. Engage in regular exercise** Regular exercise is important for general health, and can help you sleep better, so it should be included around your shift schedule and lifestyle. Keep in mind that research now shows that nighttime exercise doesn’t disrupt sleep for most people; however, it’s also important to spend time winding down before bed.
**Guideline 18. Develop strategies for sleep problems** If you’re unable to sleep, get out of bed and do something relaxing in a quiet, dimly lit environment. Try to limit screen time and clock-watching and go back to bed once you’re feeling sleepy. If sleep problems occur more than 3 times/week for several weeks in a row, seek advice from a healthcare professional.

NB: These guidelines are intended to be used as a general tool to improve shift worker sleep. For optimal use, shift workers should seek expert advice (e.g. general practitioner, sleep physician) in employing these guidelines, particularly to understand how time-dependent elements (e.g. medication use, light exposure) may require tailoring.

^*^Denotes individual guidelines that were specifically created for shift workers, with remaining guidelines representative of existing advice that has been tailored for this population.

While consensus was reached on all individual guidelines within three rounds, a small number of experts indicated that the final version of each guideline was not appropriate for inclusion as written. Across all guidelines, there were an average of 10 experts (18%) who indicated they “strongly disagreed,” “disagreed,” or “neither agreed nor disagreed*”* with the final version of the guideline being included as written. Additional detail regarding consensus outcomes for each of the individual guidelines is provided in Table 2.

## Discussion

The present study utilized a Delphi methodology to gather expert opinion regarding the applicability of current sleep hygiene guidelines for shift workers, the appropriateness of the term “sleep hygiene” in referring to such guidelines, and consequently, develop tailored sleep hygiene guidelines for shift workers. The majority of experts (71%) who participated agreed that the current sleep hygiene guidelines are not appropriate for use by shift workers, while most (84%) also agreed that tailored guidelines were required for this population. Additionally, of the alternate terms suggested by experts, most (54%) preferred the term “healthy sleep practices” for use when referring to the tailored guidelines. The tailored guidelines consist of eighteen individual guidelines (17 drafted by the research team and one new guideline developed during the Delphi rounds) and an introductory statement (also developed during the Delphi rounds). Following responses from experts regarding sleep hygiene terminology, these tailored guidelines will be referred to, in both publication and with consumers, as “Healthy Sleep Practices for Shift Workers.”

Six of the eighteen tailored guidelines provide advice that is consistent with current sleep hygiene guidelines; these were: consistently practicing a relaxing bedtime routine (guideline 4) [63], creating a cool, dark, and quiet sleep environment (guideline 8) [81–83], using bed for sleep and intimacy only (guideline 9) [63], limiting or entirely avoiding nicotine (guideline 12) [96], limiting alcohol consumption (guideline 13) [95], and engaging in regular exercise (guideline 17) [109]. The wording in some of these was altered slightly to ensure relevance for shift workers, for example “including exercise around shift schedule and lifestyle”; however, the messaging remained unchanged. The remaining twelve guidelines were either developed specifically for shift workers or were current guidelines that required significant modification to ensure their relevance.

Of the 18 guidelines, some reached consensus earlier than others. Notably, guideline 12 (on nicotine) was the only guideline to reach consensus during round 1, which may be attributed to the universal applicability of this advice (abstaining from nicotine consumption), regardless of population sleep characteristics. Several other guidelines—on sleep duration (guideline 2), sleep environment (guideline 8), alcohol consumption (guideline 13), and strategies for sleep problems (guideline 18)—reached a high level of consensus (>90%) in round 2, following modification by the research team based on round 1 feedback. Guideline 3 (on sleep scheduling), guideline 5 (on transitioning to days off), and guideline 6 (on napping) were the only guidelines that required three rounds to reach consensus. While these three guidelines address different topics, it is unsurprising that they were more contentious than others, given the difficulty in tailoring such advice for shift workers. For example, guideline 3, in relation to sleep scheduling, represents an element of the current sleep hygiene guidelines that is inappropriate for shift workers, given their inability to maintain a regular sleep schedule. As such, this guideline had to be entirely reconsidered within the context of individuals who cannot go to sleep and wake at the same time each day. This is further complicated by the vast array of shift and roster types employed across occupational contexts, and the ways in which they impact sleep and wake times. Guideline 5, on transitioning to days off, was challenging for similar reasons (i.e. a multitude of shift and roster types that lead into non-work periods). However, unlike guideline 3, which was existing advice that was tailored to shift workers, guideline 5 was one of several guidelines created specifically for this group, as current sleep hygiene guidelines do not require advice about sleep in the context of transitioning to non-work periods. Guideline 6, regarding napping, also required three rounds to reach consensus. While experts readily agreed that napping is a valuable tool for managing the fatiguing effects of shift work [35], the best way to communicate this to shift workers was challenging. For example, the duration of naps can impact outcomes (e.g. sleep inertia) and efficacy (e.g. fatigue reduction) [35], and applying this to the vast range of shift working arrangements, industries, and job roles also required consideration. Furthermore, this is another item that demonstrates the unique tailoring required for this demographic, as it contradicts some current sleep hygiene guidelines that advise against daytime napping [18].

The nature of feedback received from experts was largely dependent on the specific guideline being reviewed. However, several themes repeatedly emerged across different guidelines and questionnaire rounds. First, experts consistently requested a “softening” of the language in the guidelines (i.e. suggesting alterations in consumer behavior rather than instructing change). This was addressed by including statements such as “where possible” in many of the guidelines, for example, in guideline 10, which advises shift workers that “Exposure to bright light before bed can impact your sleep. Try to limit exposure where possible….” In addition, this feedback was addressed through the inclusion of the introductory statement. The introductory statement endeavors to recognize the unique challenges faced by shift workers, such as attempting to sleep at nontraditional times [25, 26], chronic difficulties in initiating and maintaining sleep [61], being alert and active during nighttime hours [115], and the consequent impact on a range of health [19], well-being [31], occupational [32], and safety outcomes [33]. Furthermore, the introductory statement encourages shift workers to implement an experimental approach to their sleeping habits by describing the guidelines as representative of the current evidence base, while suggesting a process of “trial and error” to determine how they can be best incorporated by each individual. Second, experts were consistent in their preference for less prescriptive guidelines. This is demonstrated by comparing the draft guidelines against the final version (Supplementary Table 1 compared to Table 6), which shows that very specific recommendations (e.g. exercise duration per week, caffeine dose per day, etc.) were removed. Experts indicated that this would allow for greater applicability across contexts (occupation, geography, etc.), whilst also considering interindividual differences. However, it is worth noting that this less prescriptive approach is the preference of experts, with additional investigation required to determine if consumers (i.e. shift workers) prefer more detailed advice (e.g. specific frequency and dose of caffeine for fatigue management).

Despite consensus being reached on all individual guidelines within three rounds, a small number of experts reported that the final version of each guideline was not appropriate for inclusion as written. Furthermore, given the broad evidence base used in developing these guidelines, certain elements are founded on unequivocal evidence (e.g. avoiding alcohol and nicotine in the context of sleep), while other evidence (e.g. the potential increased risk of an accident vs. benefit of wearing sunglasses to reduce bright light exposure during the commute home after night shift) requires further investigation. As the evidence base develops over time, the advice included in such guidelines will require updating to ensure it remains best practice.

### Guideline implementation and future directions

This study has taken an important first step in amending existing sleep hygiene guidelines to be more relevant for shift workers. To build upon this, future research must focus on the acceptability and implementation of the guidelines. Specifically, future studies should: investigate accessibility of the language used in the guidelines to ensure they are appropriately communicating key messages to shift workers as consumers; investigate shift worker attitudes towards the newly developed guidelines; ascertain the practicality of implementing the guidelines in day-to-day life; and determine the impact of tailored guideline implementation on sleep outcomes amongst shift workers. Ideally, future studies will utilize a randomized controlled trial methodology, to ensure appropriate rigor in investigating the ability of the tailored guidelines to improve sleep and well-being outcomes (i.e. sleep quantity, sleep quality, and fatigue on shift) amongst shift workers. Furthermore, future work must consider the vast range of shift and roster types employed in shift work settings. For example, certain sleep hygiene guidelines may improve sleep outcomes across certain roster types but not others, and this differentiation must be explored. Finally, future studies should investigate the appropriateness of the language used in the guidelines (e.g. does “roster” have cross-cultural relevance, or is another term more appropriate for describing work patterns?), and their ability to be accurately translated without losing context or minimizing the potential impact on shift worker sleep outcomes.

There are several contexts in which the tailored guidelines may be implemented for shift workers, following rigorous validation as outlined above. First, the tailored guidelines may be made available for shift workers as a tool to improve their general sleep health. Sourcing health information online is increasingly popular, with consumers actively seeking out information through internet searches or social media groups, particularly following diagnosis of a health condition by a professional [116, 117]. Given that the tailored guidelines represent noninvasive lifestyle and environmental adaptations, there is little concern about them being inappropriately applied, and as such, they could be easily and safely distributed online.

Occupational health and safety or fatigue management contexts are also relevant when considering the implementation of the tailored guidelines. A significant body of evidence demonstrates the occupational safety outcomes that result from poor sleep associated with shift work, including increased workplace errors, accidents, and injuries [118–121]. Beyond the impacts on the individual worker, these safety outcomes can decrease productivity and increase organizational expenditure [122, 123]. As such, employers should have a vested interest in ensuring that shift workers are able to obtain optimal sleep. The tailored guidelines represent a resource that shift work employers can easily provide to their employees, alongside other workplace health and safety resources.

The tailored guidelines may also have an indirect research benefit. In experimental sleep studies investigating behavioral interventions, sleep hygiene guidelines may be utilized as a control measure [3,4]. However, when shift workers are the cohort being investigated, using current sleep hygiene guidelines poses the risk of implementing a control measure that is either impractical or detrimental to participants. Therefore, the tailored guidelines represent a standardized control measure that can be implemented by researchers when conducting experimental studies with shift workers, which does not risk undermining the measured treatment effect.

### Strengths and limitations

In developing the tailored guidelines, the present study benefited from a high level of engagement and the timely responses of experts, which was enhanced by the ability to conduct the Delphi methodology online, allowing engagement with an international expert community. However, it was not possible to create an exhaustive list of experts, which inevitably meant that some key individuals may not have been consulted. All research team members contributed names of relevant experts, in addition to those identified through review of the literature, and experts were encouraged to share the invitation with other appropriately qualified individuals. In addition, despite using a reproduceable methodology to identify and recruit expert participants, the majority of those invited and those who participated were in either Australia or North America. While this may be where the majority of sleep and shift work experts are located, input on the guidelines from an even broader cross-section of experts in the sleep and shift work fields may have a more global impact.

It is also worth noting that expert opinion, even by consensus, can be flawed, with the tailored guidelines representative of the current best-practice recommendations. In addition, expert opinion is not always homogenous, as represented by the small proportion of expert participants and peer reviewers who did not agree with some of the wording used in the final version of the guidelines. As such, it is vital that the guidelines are revised as the evidence base on sleep, shift work, and occupational health evolves. Furthermore, one limitation of the Delphi methodology is that the research team developed the draft guidelines prior to recruitment and without the input of a larger group of experts. However, as a result of feedback review from the expert pool, the research team were able to create new guidelines or remove draft guidelines to ensure that the final set of tailored guidelines considered expert opinion, despite experts not having been involved in their initial development.

Finally, it is worth noting that this study, and the expert participants, have considered shift work in its broad context, without differentiating the multitude of shift-working systems that are utilized. It is important to stress the highly variable nature of shift work schedules, and the different ways in which shift workers respond to these schedules, particularly in relation to their sleep health. As such, the future research mentioned above, which will aim to investigate the efficacy of the tailored guidelines at improving shift worker sleep, must attempt to include as wide a variation of shift work schedules and individual characteristics (e.g. age, gender, health status, etc.) as possible.

## Conclusion

This study represents the use of a Delphi methodology to develop “Healthy Sleep Practices for Shift Workers,” amended sleep hygiene guidelines for this sleep-vulnerable population. Totaling eighteen individual guidelines and an introductory statement, with revised terminology, these tailored guidelines could provide shift workers with evidence-based, relevant advice on adapting lifestyle and environmental factors in order to optimize their sleep quantity and quality. Beyond their implementation for shift workers seeking to improve their sleep health, the tailored guidelines have additional occupational and research applications. Future research should focus on investigating shift worker understanding and acceptance of the tailored guidelines and determining their ability to improve sleep outcomes among shift workers.

## Supplementary Material

zsad182_suppl_Supplementary_Table_S1

## Data Availability

The de-identified data that supports the findings of this study is available from the corresponding author upon reasonable request.
