# Supplementary material for: Healthy sleep practices for shift workers: consensus sleep hygiene guidelines using a Delphi methodology
Source: Sleep. 2023 Jul 10;46(12):zsad182. doi: 10.1093/sleep/zsad182 (PMC10710992; doi:10.1093/sleep/zsad182)
Supplement: zsad182_suppl_Supplementary_Table_S1 [file zsad182_suppl_supplementary_table_s1.docx]

**Healthy Sleep Practices for Shift Workers:**

**Consensus Sleep Hygiene Guidelines using a Delphi Methodology**

Alexandra E. Shriane^1^, Dr Gabrielle Rigney^1^, Professor Sally A. Ferguson^1^, Dr Yu Sun Bin^2^, Dr Grace E. Vincent^1^

^1^ Appleton Institute, School of Health, Medical and Applied Sciences, Central Queensland University, Adelaide, South Australia, Australia.

^2^ University of Sydney, Sydney, New South Wales, Australia.

**Corresponding Author:**

Alexandra Shriane, School of Health, Medical and Applied Sciences, Appleton Institute, CQUniversity, 44 Greenhill Road, Wayville, SA 5034; alex.shriane@cqumail.com

**Supplementary Table 1. Draft sleep hygiene guidelines for shift workers as developed by the research team**

| **Guideline No.** | **Guideline (draft)** |
| --- | --- |
| Introductory Statement | N/A – developed following Round 1 feedback |
| 1. Sleep Prioritisation | Prioritise your sleep and aim to wake up as close to the start of your shift time as possible. This may involve rescheduling social activities or delegating household tasks to others |
| 2. Sleep Duration | Obtain 7-9 hours of sleep per 24 hours. This may be achieved as one single sleep period, or through multiple shorter sleeps. Keep in mind this is total time spent asleep, not just time in bed |
| 3. Sleep Scheduling | Develop a sleep routine based on shift type and maintain this routine as much as possible. For example, sleep from 9pm-5am when working day shifts, and from 10am-6pm when working night shifts |
| 4. Bedtime Routine | Develop a relaxing bedtime routine, as use it as much as possible, ideally in the 1-2 hours before bed. For example, switch off electronic devices, have a warm bath/shower, and engage in relaxing activities (e.g., reading, meditating) |
| 5. Transition to Days Off | When transitioning to days-off, particularly after working late/night shifts, aim to have a short sleep in the morning, wake up by the middle of the day, get some sunlight shortly after waking, then head back to bed in the early evening for a full night’s sleep |
| 6. Napping | Use strategic napping, aiming for short refreshing naps (15-20 minutes), or longer, more restorative naps (90 minutes) |
| 7. Sleep Inertia | N/A – developed following Round 1 feedback |
| 8. Sleep Environment | Aim for ‘cave like’ bedroom conditions: a cool room (16-24 degrees Celsius with adequate ventilation), a dark room (block out as much light as possible with appropriate window furnishings or eye masks), and a quiet room (block out as much noise as possible by closing doors and windows, using ear plugs, and switching off devices). |
| 9. Bed Use | Use your bed for sleep and intimacy only. If napping at home, nap in your bedroom to ensure the best sleep quality possible. |
| 10. Light | Seek out bright light (e.g., sunlight, bright artificial light) before and during your shift, but avoid these bright light sources before bedtime where possible, opting for dimly lit environments or wearing sunglasses. This includes limiting exposure to blue light (e.g., mobile phones, computers, televisions) in the 1-2 hours before bed. |
| 11. Caffeine | Consider the following when it comes to caffeine: caffeine can help to manage feelings of fatigue before and during shift, caffeine effects can last up to 8 hours, so only use it within 8 hours of bed when really needed, don’t consume more than 400mg caffeine/24 hours, don’t consume more than 200mg in a single serving. |
| 12. Nicotine | Avoid nicotine entirely, or limit nicotine intake in the 6 hours before bed. |
| 13. Alcohol | Consider the following when it comes to alcohol: avoid alcohol in the 4 hours before bed, alcohol may induce sleepiness, but will negatively affect sleep quality, consume no more than 10 standard alcoholic drinks per week, and no more than 4 per day. |
| 14. Medication Use | Medications that induce sleep (i.e., sleeping tablets) should only be used under the direction of a health care professional, and ideally, only for short-term relief of sleep problems. |
| 15. Food Intake | Consume a healthy, balanced diet. This involves consuming from the five food groups each day – vegetables, fruits, grains, proteins, and dairy/dairy alternatives. When eating on shift, particularly night shift, opt for smaller, lighter meals and avoid those high in sugar or saturated fats. |
| 16. Fluid Intake | Consider your food and fluid intake in the 1-2 hours before bed. Eating too close to bedtime can cause indigestion, while too much fluid intake can lead to sleep disturbances to use the toilet. |
| 17. Exercise | Engage in regular exercise as per the following: 2.5-5.0 hours/week of moderate intensity activity OR, 1.25-2.5 hours/week of vigorous activity AND, muscle-strengthening activity 2 days/week. Keep in mind that research now shows that exercise before bed does not disrupt sleep, however, it’s important to spend 1-2 hours before bed winding down and avoiding bright light where possible. |
| 18. Sleep Problems | If you’re unable to fall asleep within 20-30 minutes of trying, get out of bed and do something relaxing in a quiet, dimly lit environment. Try to limit screen time and clock-watching, and instead opt for something relaxing, like reading or meditating. If sleep problems continue more than one night a week, for several weeks in a row, seek advice from a healthcare professional. |

NB: Draft guidelines as presented in Supplementary Table 1 encompass the following themes, with examples of supporting evidence for such themes: ensuring sufficient sleep duration [54, 78, 79, 80-82], attempting to sleep and wake at the same time/s [55, 68, 83], engaging in a relaxing bedtime routine [56, 84-86], transitioning to days off work [87-92], napping for fatigue management [57, 93-99], maintaining a comfortable sleep environment [58-60, 100-102], managing exposure to light [108-113], managing caffeine consumption [7, 61, 114], limiting alcohol and nicotine [62, 63, 115, 116], considering medication use [64, 118-121], considering food and fluid intake [65, 122-125], engaging in regular exercise [66, 126, 127], and managing sleep problems [67, 103, 104].
